# Sleep and cognition in Bipolar Disorder in full or partial remission

**DOI:** 10.1192/j.eurpsy.2024.905

**Published:** 2024-08-27

**Authors:** K. T. Svee, H. Kallestad, G. Morken, T. I. Hansen, A. Engum

**Affiliations:** ^1^Division of Mental Healthcare, St. Olavs Hospital; ^2^Department of Mental Health, Norwegian University of Science and Technology, NTNU; ^3^St. Olavs Hospital, Trondheim, Norway

## Abstract

**Introduction:**

Cognitive impairment in Bipolar Disorder (BD) is frequent and is associated with reduced function in several areas. Close to half of the patients with BD have persistent cognitive dysfunction. The causes of cognitive impairments and factors associated with normal cognitive function are not clearly described.

Some preliminary evidence links sleep disturbances and cognition impairment in BD. A limited number of studies have investigated the link between sleep and cognitive function in BD using objective measures.

**Objectives:**

We aim to investigate associations between sleep and objective and subjective cognitive function in patients with BD in full or partial remission.

**Methods:**

This is a cross-sectional study. The participants will be 90 adults meeting criteria for DSM 5 BD type 1 or type 2 in full or partial remission. Participants are recruited from psychoeducational groups for BD and from a specialist outpatient clinic.

Diagnoses are set with SCID-5 and are confirmed in a consensus meeting with at least two psychiatrists and/or specialists in psychology. Symptoms of depression and mania are measured with Montgomery Asberg depression rating scale (MADRS) and Young Mania Rating Scale (YMRS). Sleep is measured subjectively with Insomnia Severity Index (ISI) and objectively with actigraphs which participants wear on their non-dominant hand for ten days. Subjective cognition is measured with Cognitive Complaints in Bipolar Disorder Rating Assessment (COBRA). Participants undergo neurocognitive testing with a self-administered validated web-based neuropsychological test platform. The testing is carried out in the participant’s home on their smart phones. The tests include measures of learning, storing, recalling, and recognizing visual and verbal information, working memory and reaction time. Normal cognitive function is defined as scores within or above mean on all cognitive subtests. The test-platform has been validated.

We will use descriptive statistics to examine distribution of demographic characteristics. We will test for correlations between sleep factors and subjective and objective measures of cognitive function.

**Ethics:**

The Regional Committees for Medical and Health research ethics approved the study.

**Results:**

Results will be presented at the conference. So far, 74 out of 90 participants have been included.

**Conclusions:**

We anticipate that normal sleep may be associated with good cognitive functioning. The findings of this study could offer supplementary insights into BD heterogeneity and potential treatment targets.

Abbreviations: SCID-5, Structured Clinical Interview for DSM-5

**Disclosure of Interest:**

None Declared

